# SP-A and SP-D: Dual Functioning Immune Molecules With Antiviral and Immunomodulatory Properties

**DOI:** 10.3389/fimmu.2020.622598

**Published:** 2021-01-19

**Authors:** Alastair Watson, Jens Madsen, Howard William Clark

**Affiliations:** ^1^Clinical and Experimental Sciences, Faculty of Medicine, Southampton General Hospital, University of Southampton, Southampton, United Kingdom; ^2^Southampton NIHR Respiratory Biomedical Research Centre, Southampton General Hospital, Southampton, United Kingdom; ^3^Birmingham Medical School, University of Birmingham, Birmingham, United Kingdom; ^4^Neonatology, EGA Institute for Women’s Health, Faculty of Population Health Sciences, University College London, London, United Kingdom; ^5^NIHR Biomedical Research Centre, University College London Hospital (UCLH), University College London (UCL), London, United Kingdom

**Keywords:** surfactant protein A, surfactant protein D, recombinant fragment of human SP-D (rfhSP-D), inflammation, immunoregulation, coronavirus disease 2019, severe acute respiratory syndrome coronavirus 2 (2019-nCoV), therapeutic

## Abstract

Surfactant proteins A (SP-A) and D (SP-D) are soluble innate immune molecules which maintain lung homeostasis through their dual roles as anti-infectious and immunomodulatory agents. SP-A and SP-D bind numerous viruses including influenza A virus, respiratory syncytial virus (RSV) and human immunodeficiency virus (HIV), enhancing their clearance from mucosal points of entry and modulating the inflammatory response. They also have diverse roles in mediating innate and adaptive cell functions and in clearing apoptotic cells, allergens and other noxious particles. Here, we review how the properties of these first line defense molecules modulate inflammatory responses, as well as host-mediated immunopathology in response to viral infections. Since SP-A and SP-D are known to offer protection from viral and other infections, if their levels are decreased in some disease states as they are in severe asthma and chronic obstructive pulmonary disease (COPD), this may confer an increased risk of viral infection and exacerbations of disease. Recombinant molecules of SP-A and SP-D could be useful in both blocking respiratory viral infection while also modulating the immune system to prevent excessive inflammatory responses seen in, for example, RSV or coronavirus disease 2019 (COVID-19). Recombinant SP-A and SP-D could have therapeutic potential in neutralizing both current and future strains of severe acute respiratory syndrome coronavirus 2 (SARS-CoV-2) virus as well as modulating the inflammation-mediated pathology associated with COVID-19. A recombinant fragment of human (rfh)SP-D has recently been shown to neutralize SARS-CoV-2. Further work investigating the potential therapeutic role of SP-A and SP-D in COVID-19 and other infectious and inflammatory diseases is indicated.

## Introduction

Surfactant proteins A (SP-A) and D (SP-D) are essential innate immune molecules with important roles in lung health (1–3). These work to both neutralize and enhance the clearance of pathogens while modulating the inflammatory response (4, 5). SP-A and SP-D play key roles in keeping the lungs in a non-inflamed and infection-free homeostatic state to ensure efficient gaseous exchange. In this review we focus on the dual roles of SP-A and SP-D in immunoregulation and anti-viral defense and in particular their role in protecting against immune-mediated pathophysiological processes following viral infection. Furthermore, we discuss the potential of recombinant versions of these proteins as prophylactic treatments for infectious and inflammatory diseases, ranging from neonatal chronic lung disease to coronavirus disease 2019 (COVID-19).

## Pulmonary Surfactant and SP-A and SP-D

Pulmonary surfactant is an important lipoprotein complex of the lung lining made of 90% lipids and 10% proteins. Surfactant is produced predominantly by alveolar type 2 cells and forms a mobile-liquid phase which covers the alveolar epithelium to facilitate breathing by reducing surface tension at end-expiration and preventing alveolar collapse (6, 7). Surfactant proteins B (SP-B) and C (SP-C) are small hydrophobic peptides of 14 kDa and 6 kDa, respectively. These are involved in the packaging and recycling of surfactant as well as contributing to its biophysical properties. Contrastingly, surfactant protein A (SP-A) and surfactant protein D (SP-D) are large, soluble, hydrophilic proteins which are expressed on most mucosal surfaces and have key overlapping and distinct roles in innate immunity and immunological homeostasis of the lung.

**Figure 1:**
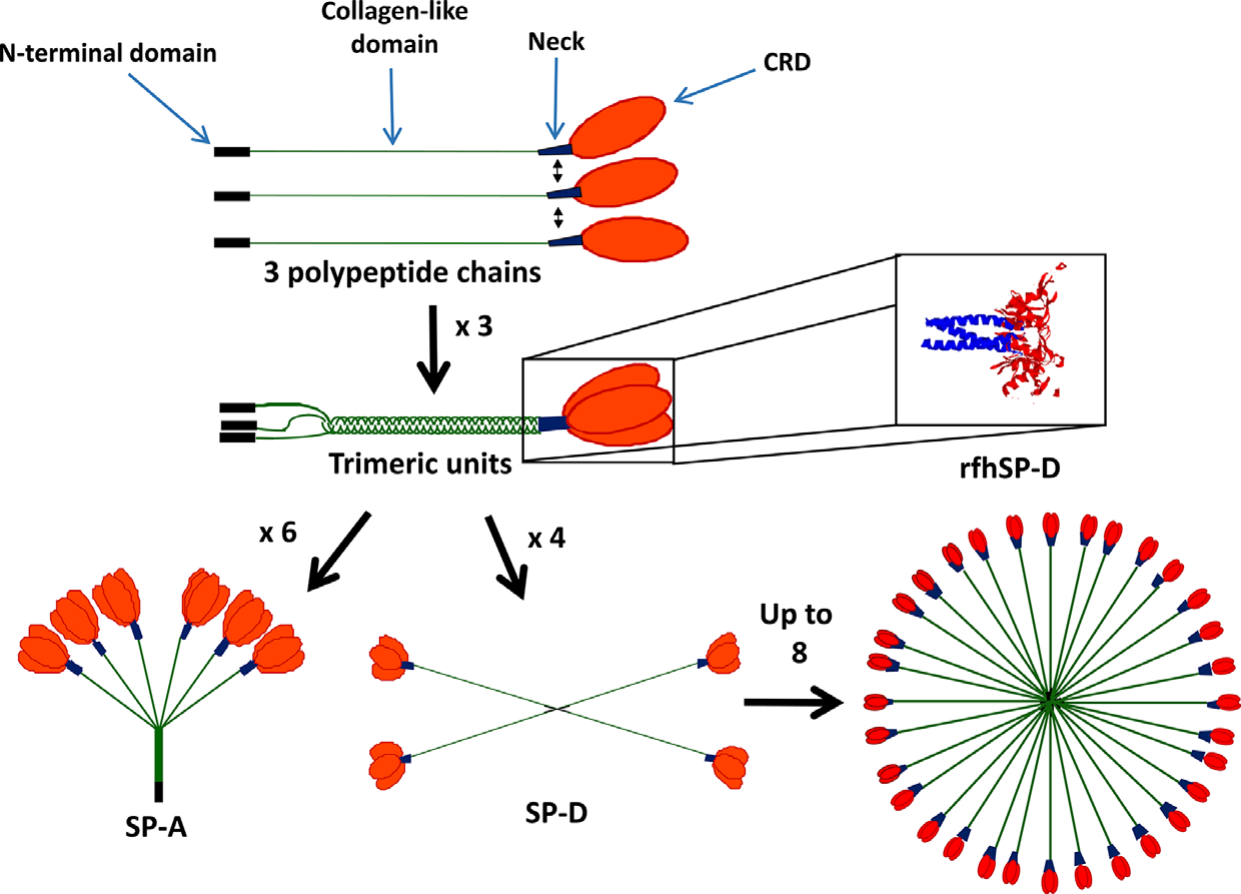
Structure of surfactant proteins A (SP-A) and SP-D. SP-A and SP-D contain four domains: the N-terminal domain (black), collagen-like domain (green), neck region (blue) and carbohydrate recognition domain (CRD) (red). SP-A and SP-D form functional trimers and can then further oligomerize into an octadecameric-like structure for SP-A and a dodecameric cruciform-like structure which can further assemble into ‘stellate multimers’ for SP-D. Also shown is the crystal structure of the recombinant fragment of human SP-D (rfhSP-D) (8). rfhSP-D is formed of the CRD, neck and 8x Gly Xaa Yaa repeats of the collagen-like region.

SP-A and SP-D form functional trimeric units, consisting of four domains, a C-terminal carbohydrate binding domain (CRD), an α-helical coiled-coil neck, a collagenous domain and an N-terminal domain (**Figure 1**). SP-A and SP-D are termed collectins as they contain **col**lagen and are functional (group III) **lectins**, which bind carbohydrates in a calcium-dependent manner using their CRD. While the SP-D trimer is a homotrimeric unit, SP-A is formed of two gene products, SP-A1 and SP-A2, and some functional differences have been described between these two molecules (9). Through interaction of their N-terminal domains, these trimeric units oligomerize into an octadecameric-like structure for SP-A, which is similar to a bunch of flowers, and a dodecameric cruciform-like structure which can further assemble into ‘stellate multimers’ for SP-D (**Figure 1**) (10). This multimerization enhances the overall avidity of binding to carbohydrate targets and enhances their capacity for pathogen agglutination.

Recombinant trimeric fragments of human SP-A (rfhSP-A) and D (rfhSP-D) have been produced and consist of the CRD and trimerizing neck regions and a collagenous stalk consisting of 8 x Gly-Xaa-Yaa repeats. These lack the capacity to agglutinate pathogens but maintain many of the anti-pathogenic and immunomodulatory functions of the native proteins. Furthermore, they have potential for development into therapeutics for a variety of inflammatory and infectious lung diseases (11–15).

## Anti-Viral Functions of SP-A and SP-D

SP-A and SP-D bind to and neutralize a number of different viruses (14). Their importance in protecting the lung against viral infections has been demonstrated by the increased susceptibility of SP-A and SP-D knockout mice to influenza A virus and respiratory syncytial virus (RSV) infection and viral-mediated inflammation (16–21).

### Influenza A Virus

As compared with wildtype mice, both SP-A and SP-D knockout mice have increased susceptibility to influenza infection with an increase in viral load, infiltration of inflammatory cells, production of inflammatory cytokines and immunopathology (17, 18, 22, 23). SP-D neutralizes influenza virus through interaction with high mannose oligosaccharides in close proximity to the hemagglutinin (HA) binding site, preventing binding to the sialic acids on the host cell (24). Administration of exogenous SP-D into the lung of SP-D knockout mice decreases viral load and reduces neutrophil infiltration, as well as levels of inflammatory cytokines within the lung, including tumor necrosis factor alpha (TNF-α) and interleukin (IL)-6. Similarly, rfhSP-D has been shown to neutralize and downregulate pro-inflammatory cytokines *in vitro* including TNF-α, interferon (IFN)-α, IFN-β, interleukin (IL)-6, and regulated on activation normal T-cell expressed and secreted (RANTES), upon influenza infection of a basal epithelial cell line (25, 26). Comparatively, SP-A occupies the HA binding site through its own salicylic acid, found naturally on the asparagine 187 residue of the CRD. This prevents binding of influenza to salicylic acids on the host cell (27). Alongside increased viral loads, SP-A knockout mice infected with influenza develop epithelial injury and higher levels of IL-6, macrophage inflammatory protein 2 (MIP-2) and macrophage and neutrophil infiltration (20, 23). Treatment with exogenous SP-A decreases influenza infection and the production of inflammatory cytokines including TNF-α, IL-6 and IFN-γ (28).

Although both SP-A and SP-D have overlapping roles in neutralizing influenza virus, they also likely have distinct roles *in vivo*. For example, SP-A but not SP-D has been shown to opsonize influenza and enhance phagocytosis by rat macrophages (29). A recent study further demonstrated that native human SP-A reduced infection of an epithelial cell line by pH1N1 and H3N2 strains of influenza *in vitro* (26). In this paper a rfhSP-A, which was composed of the CRD and neck without a collagen stalk, interacted with neuraminidase and matrix protein 1 in a calcium-dependent manner. However, it was shown that this fragment enhanced influenza infection as well as expression of inflammatory cytokines TNF-α, IFN-α, IFN-β, IL-12, IL-6, and RANTES, contrasting to the native molecule. This opposing effect of the SP-A fragment is interesting and could be explained by its expression in an *Escherichia coli* strain, which lacks the capacity to add N-linked glycosylations to the expressed protein. This fragment, therefore, lacks the asparagine 187 residue which is known to be important for influenza A neutralization, which may mean that the SP-A fragment interacts with influenza through a different mechanism. Alternatively, the trimeric structure of this molecule as opposed to the octadecameric structure of the native protein could impact the ability of this molecule to neutralize or aggregate influenza and allow enhancement of epithelial cell infection. Further work elucidating this difference between native SP-A and SP-D and their recombinant fragments in *ex vivo* and epithelial-macrophage co-culture models will be important to understand their role in the influenza infected lung and the potential for therapeutic use (30).

### Respiratory Syncytial Virus

RSV is the leading cause of lower respiratory tract infection in infants worldwide and is characterized by an excessive immune response with a T helper (Th)2 bias (31, 32). SP-A knockout mice have an enhanced susceptibility to RSV, with increased viral loads, infiltration of immune cells and production of inflammatory cytokines including TNF-α, IL-6, and IL-1β (18). *In vitro* work has demonstrated the capacity of SP-A to neutralize RSV through binding the fusion (F) protein (33). Furthermore, administration of native SP-A to SP-A knockout mice both prevented RSV infection and decreased total bronchoalveolar lavage (BAL) inflammatory cell numbers (33). However, another *in vitro* study found native human SP-A to bind to the attachment protein (G) of RSV and enhance the update of RSV by Hep-2C cells, potentially *via* its N-terminal domain (33). More recent research confirmed the role of native SP-A in neutralizing RSV, but found trimeric rfhSP-A lacking the N-terminal domain to be more efficacious (11). Further work is, therefore, needed to investigate the importance of the N-terminal domain in mediating RSV infection.

SP-D interacts with RSV through both the fusion (F) and attachment (G) proteins (19). SP-D knockout mice also have increased levels of inflammatory cytokines following RSV infection including TNF-α, IL-1β, IL-6, and MIP-2. Administration of either exogenous native human SP-D or rfhSP-D into the lung neutralizes RSV *in vivo* (19, 35). However, despite these early studies, there has been no recent work demonstrating the importance of SP-D treatment in preventing RSV-induced inflammation and immunopathology. Further work will be key in assessing the potential of recombinant SP-D as a therapeutic in RSV infections.

Parainfluenza is a virus related to RSV which commonly infects the elderly and immunocompromised (36). However, the role of SP-A and SP-D in modulating parainfluenza infection and parainfluenza-mediated immunopathology is yet to be described. SP-D has, however, been reported to inhibit hemagglutination activity of Sendai virus, the related murine parainfluenza virus (37). Further studies on the direct interaction of SP-A and SP-D with parainfluenza are needed.

### Coronaviruses

SP-A and SP-D play roles in modulating coronavirus infection and early work demonstrated their ability to bind human coronavirus 229E (HCoV-229E) virions and prevent infection of human bronchial epithelial cells (38). Notably SP-D was more efficient than SP-A at neutralizing HCoV-229E virions to prevent human bronchial epithelial cell line infection. However, SP-A, but not SP-D was demonstrated to reduce infection of human alveolar macrophages (39).

SP-D has been shown to bind the heavily glycosylated SARS-coronavirus (CoV) spike (S) protein (39). Furthermore, pre-incubation of SP-D with SARS S-protein increases binding of S-protein to DCs, but not macrophages or a kidney epithelial cell line (40). Plasma levels of SP-D have been found to be elevated in severe acute respiratory syndrome (SARS)-related pneumonia, potentially through leakage from the damaged lung into the blood (40). Furthermore, recent studies have shown that COVID-19 patients who went on to develop macrophage activation syndrome had significantly higher serum levels of SP-D on admission and that SP-A and SP-D serum levels correlated with more severe COVID-19 disease (41, 42). Thus, early work suggests a role for SP-D in SARS-CoV-2 infection, which may modulate infection and the pathologic host inflammatory response. The level of SP-D in the lung and potential role in SARS infection immunopathology, therefore, merit consideration. Equally, the potential role of serum SP-D levels as a potential biomarker in SARS-related pneumonia warrants further investigation.

The severity of COVID-19 disease resulting from the current SARS-CoV-2 pandemic is in part related to aberrant host-inflammatory responses (43, 44). SP-A and SP-D could play roles in modulating inflammation and binding to and neutralizing SARS-CoV-2 through interaction with the spike protein which is also heavily glycosylated (43) rfhSP-D has recently been shown to compete with ACE-2 for binding of the S1 spike protein subunit of SARS-CoV-2. Furthermore, rfhSP-D reduced infection of a cell line by SARS-CoV-2 from clinical samples (45, 46). The impact of rfhSP-D on the inflammatory response is still to be demonstrated. However, rfhSP-D may have therapeutic potential in treatment of COVID-19. The role of SP-A and SP-D in COVID-19 disease now needs to be fully determined in both the serum and lung in *in vivo* and *ex vivo* models. Furthermore, the potential for recombinant versions of SP-A and SP-D to modulate SARS-CoV-2 infection and immunopathology warrants investigation.

### Human Immunodeficiency Virus

Outside the lungs, SP-A and SP-D are expressed within the urogenital tract and most other extra-pulmonary mucosal surfaces (47–49). SP-A and SP-D play dual roles in HIV infection and pre-incubation with HIV both neutralizes the virus to prevent infection of a cluster of differentiation (CD)4+ T cell line (PM1 cells), as well as enhance infection of immature monocyte-derived dendritic cells (IMDDCs) and subsequent transfer to T cells (50, 51). SP-A binds to glycoprotein (gp)120 and blocks its interaction with both CD4 and Dendritic Cell-Specific Intercellular adhesion molecule-3-Grabbing non-integrin (DC-SIGN). Similarly, SP-D also binds gp120 and gp41 and blocks the interaction of gp120 with DC-SIGN. However, there have been conflicting reports around the capacity of SP-D to disrupt the binding of gp120 to CD4 (50, 52). The mechanism by which SP-A and SP-D enhances uptake into DCs is still not fully characterized. This could be through interaction of the HIV-bound collectin with a host cell receptor and agglutination of the virus to enhance uptake. Upon occupation of the collectin CRD, SP-A and SP-D bind numerous host cell receptors, principally through their N-terminus (**Table 1**). A rfhSP-D molecule lacking the N-terminal domain could therefore be advantageous in neutralizing HIV without agglutination or interacting with dendritic cell (DC) receptors. Pandit et al. demonstrated the function of rfhSP-D in neutralizing HIV to prevent T cell infection. However, the effects of HIV pre-incubation with rfhSP-D on DC uptake and subsequent transfer to T cells was not determined (52). Dodagatta-Marri et al. demonstrated that rfhSP-D inhibited HIV-1 transfer to activated peripheral blood mononuclear cells when pre-incubated with a human embryonic kidney cell line (60). They further demonstrated that rfhSP-D was able to interact with DC-SIGN as well as compete with DC-SIGN to interact with gp120. Further work directly comparing the interaction with HIV of both native SP-A and SP-D and their recombinant fragments would be useful to determine the structure-function relationships affecting their capacity to neutralize HIV and modulate viral transfer to T cells.

Alongside neutralization of HIV, SP-D has been shown to play important roles in modulating HIV-mediated inflammation. *In vitro* treatment of Jurkat T cells with SP-D upon HIV infection decreased expression levels of IL-2, IFN-γ, vascular endothelial growth factor (VEGF), IL-1α, and TNF-α. Similarly, treatment of peripheral blood mononuclear cells (PBMCs) with SP-D during HIV infection decreased IL-2, IFN-γ, VEGF, IL-6, monocyte chemoattractant protein-1 (MCP-1) and IL-1β (52). However, despite the promising potential of SP-A and SP-D for modulating HIV infection and HIV mediated inflammation, the full impact of SP-A and SP-D on HIV infection and immunopathology *in vivo* and in the human disease is yet to be determined.

### Other Current and Emerging Viruses

SP-A and SP-D are broadly selective innate immune proteins and thus are likely to play key roles in modulating infection and inflammation mediated by other viruses. SP-A, but not SP-D, binds to human papillomavirus 16 (HPV16) pseudovirions and enhances their uptake by RAW267.4 macrophages and clearance *in vivo* (91). SP-A also binds herpes simplex virus (HSV) infected cells, as well as HSV virions through its Asn187 carbohydrate moiety to enhance phagocytosis by rat macrophages (92, 93). Further work is needed to characterize the mechanism of SP-A interactions with HPV and HSV and its role in modulating inflammatory responses to these viruses (94).

SP-D but not SP-A has been demonstrated to bind to the Ebola virus in a calcium-dependent manner through its CRD (95). However, pre-incubation of human SP-D (but not rfhSP-D) with the Ebola virus enhanced infection of Vero cells; this could be mediated through membrane receptor interactions with the collectin N-terminal domain. The potential of trimeric rfhSP-D in therapeutic modulation of the aberrant pro-inflammatory cytokine release by monocytes and macrophages in Ebola virus infection is as yet untested (96), as it is for other currently emerging viruses.

## Immunomodulatory Functions of SP-A and SP-D

**Figure 2:**
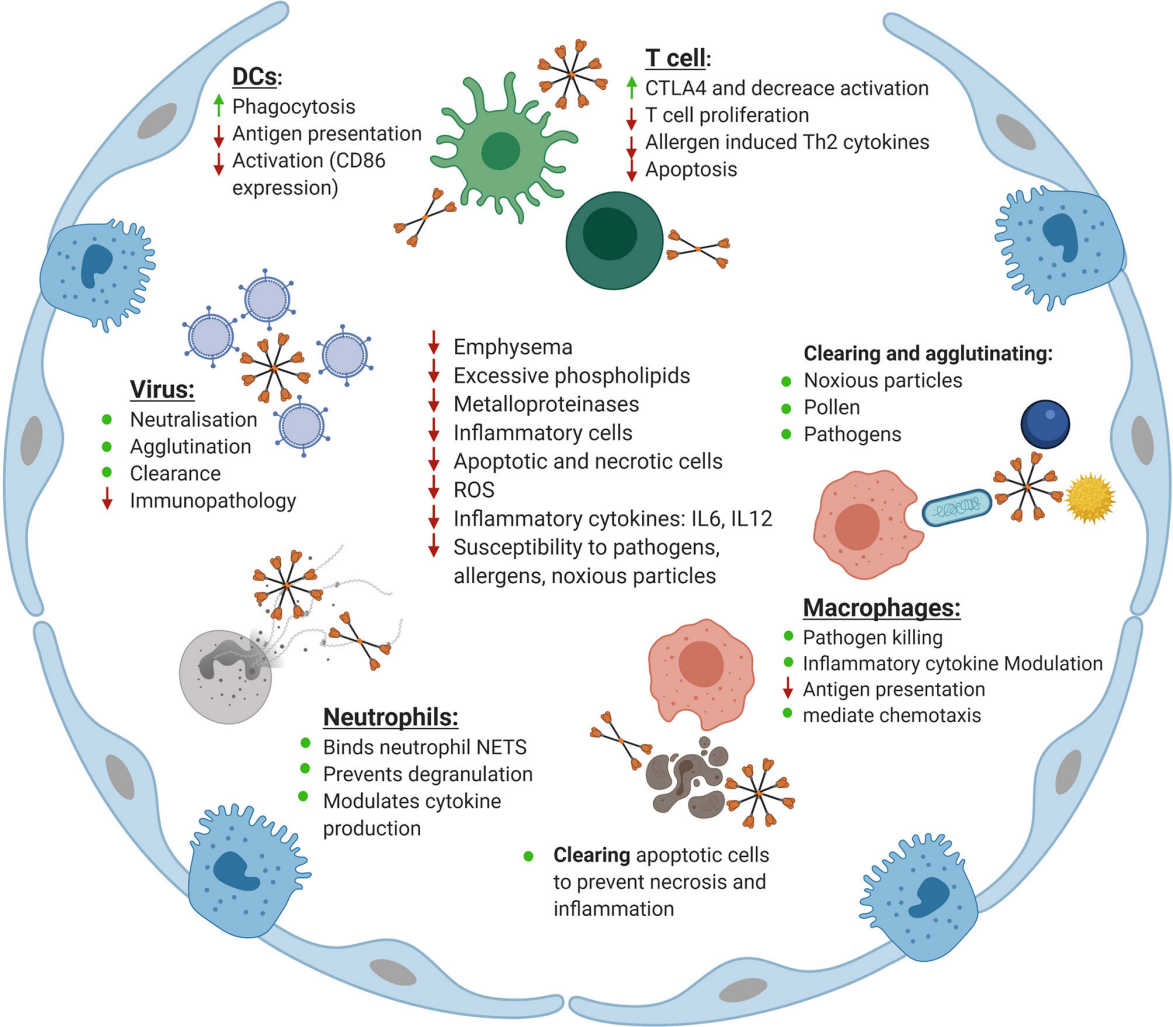
Maintenance of homeostasis in the lung by surfactant protein D (SP-D). Shown is an overview of the roles of SP-D in the lung. Indicated is the role SP-D plays in neutralizing, agglutinating and clearing viruses as well as reducing the inflammatory response upon infection with influenza A virus, respiratory syncytial virus (RSV), and human immunodeficiency virus (HIV). The role of SP-D in enhancing phagocytosis by dendritic cells (DCs) while simultaneously reducing antigen presentation and activation of co-stimulatory markers is indicated. Also shown is the role of SP-D in keeping T cells in a hyporesponsive state to increase CTLA4 expression, reduce T cell proliferation, reduce allergen induced Th2 cytokine production and modulate apoptosis. The role of SP-D in clearing and agglutinating noxious particles, pollen and pathogens is indicated. Similarly, the role of SP-D in enhancing macrophage-mediated pathogen killing, modulating inflammatory cytokine production by macrophages and macrophage chemotaxis and reducing antigen presentation is displayed. Also shown is the role of SP-D in clearing apoptotic and necrotic cells in the lung as well as its interaction with neutrophils in binding to neutrophil NETS, and eosinophil extracellular traps, preventing degranulation and modulating cytokine production. Finally, the role of SP-D and rfhSP-D in correcting the phenotype of the SP-D knockout mouse is indicated, specifically their role in decreasing emphysema, excessive phospholipid production, decreasing inflammatory cell and apoptotic and necrotic cell numbers, decreasing the level of reactive oxygen species (ROS), decreasing inflammatory cytokines including IL-6 and IL-12 and decreasing the susceptibility of SP-D knockout mice to pathology as a result of challenge with pathogens, allergens and noxious particles. Adapted from “Alveolar Epithelium (Comparison)”, by BioRender.com (2020). Retrieved from https://app.biorender.com/biorender-templates.

SP-A and SP-D are key defense molecules which neutralize a range of viruses. However, a plethora of *in vitro* and *in vivo* studies have demonstrated SP-A and SP-D as key players in directly modulating the innate and adaptive immune system independent of infection (**Figure 2**). Through these mechanisms, SP-A and SP-D could be important in balancing the inflammatory response to prevent immune-mediated pulmonary pathology, an important feature of influenza and SARS-related viral pneumonia, as well as RSV induced bronchiolitis (97, 98).

### The Phenotype of SP-A and SP-D Knockout Mice

The importance of SP-A and SP-D in maintaining lung homeostasis is highlighted by the inflammatory phenotype of the knockout mice. SP-D deficient mice have increased infiltration of macrophages and activated T cells, the appearance of foamy macrophages with an excessive level of apoptotic and necrotic macrophages and alveolar type II cell hyperplasia in the airways. They also have excessive levels of phospholipids, overproduction of reactive oxygen species (ROS) and increased levels of lung IL-6, IL-12 and metalloproteinases (MMPs). By the age of three weeks, these mice already show signs of a progressive emphysema-like phenotype with loss of alveolar septation and the appearance of foamy macrophages (99–101). This phenotype can be resolved upon therapeutic treatment with rfhSP-D, which corrects the emphysema and decreases the number of apoptotic and necrotic alveolar macrophages, excess phospholipid production and alveolar type II cell hyperplasia (100, 102).

Allergy mouse models have also demonstrated the role of SP-D in immunoregulation with increased IL-13 levels and BAL eosinophils upon ovalbumin sensitization in SP-D knockout mice (103). Similarly, as compared with wildtype mice, SP-D deficient mice exposed to *Aspergillus fumigatus* allergen have enhanced CD4 T cells numbers, IgG1 and IgE immunoglobulins and Th2 cytokines, with a decrease in IFN-γ (104–106). This highlights the potential role of SP-D in preventing Th2 inflammatory skewing, which has been shown to be important in immune evasion by viruses such as RSV (107). SP-D knockout mice also have an enhanced susceptibility to cigarette smoke-induced airway inflammation with influx of alveolar macrophages, secretion of chemokine (C-C motif) ligand 3 (CCL3) and IL-6 and upregulation of ceramide genes; rfhSP-D alleviates this *in vivo* phenotype and attenuates cigarette smoke induced human epithelial cell apoptosis (108).

By contrast, SP-A knockout mice kept in sterile vivarium conditions have relatively unaltered lungs and normal lung function. However, they also exhibit increased susceptibility to a range of pathogens and enhanced inflammatory responses to pathogen challenge, showing, for example higher levels of TNF-α and nitric oxide metabolites upon intranasal delivery of lipopolysaccharide (LPS); this is corrected upon therapeutic treatment with exogenous SP-A (18, 22, 109–114). SP-A deficient mice also show excessive inflammation following allergen challenge with marked hyper-eosinophilia and increased IL-5 and IL-13 upon challenge with *Aspergillus fumigatu*s allergens (106). This excessive inflammatory response in SP-A and SP-D knockout mice to pathogen-associated molecular patterns (PAMPs) on allergens and in infection, highlights their critical role in maintaining the lung in a non-inflamed condition, preserving homeostasis and facilitating gas exchange. This may be crucial to both prevent excessive inflammation and reduce viral-mediated lung pathology in chronic lung diseases.

### Roles of SP-A and SP-D as Innate Immune Scavenger Receptors

#### Agglutination of Bacteria and Fungi and Their Components

SP-A and SP-D help preserve lung homeostasis by acting as innate immune scavenger receptors (115, 116). Their importance in binding to and clearing an array of different gram negative and positive bacteria and fungi, as well as their components, has been widely reported (116–118). This can occur through interacting with LPS through binding to terminal monosaccharides and lipid A. Binding pathogens by SP-A and D leads to their agglutination, while also directly enhancing uptake by macrophages and neutrophils through interactions mediated by various receptors (**Table 1**) (5, 116, 117, 119, 120). Comparative to the native oligomeric proteins, fragments of SP-A and SP-D are trimeric and lack the capacity to agglutinate bacteria. SP-A and SP-D can also modulate receptor expression on macrophages including mannose receptor, an important receptor for mediating phagocytosis (121, 122). Alongside enhancing clearance of pathogens, SP-A and SP-D also enhance macrophage-mediated killing of bacteria through increasing the production of nitric oxide as well as directly increasing membrane permeability of gram negative bacteria to inhibit their growth (123–126). However, *Bordetella pertussis* lipopolysaccharide resists the bactericidal effects of pulmonary surfactant protein A and the ability of SP-A to bind and aggregate the bacteria; this protective effect was lost in LPS mutants which lacked the terminal trisaccharides, suggesting that *B. pertussis* has evolved a mechanism which shields against the anti-bacterial function of SP-A (124). SP-A has been shown to enhance TNF-α and nitric oxide mediated killing of Bacillus Calmette-Guerin by rat macrophages (127). These roles could be particularly important for the prevention of secondary bacterial infection and resolution of inflammation following viral infection (128).

**Table 1:** The interaction of surfactant proteins A (SP-A) and SP-D with host cell surface proteins, soluble proteins, and receptors.

Protein	Target Cell/Function	Collectin	Reference
SPR-210 (Myosin 18A/CD245)	Monocytes, macrophages, T-cells, type II epithelial cells	SP-A	(53–57)
CD14	Myeloid lineage cells	SP-A and SP-D	(58, 59)
DC-SIGN	Macrophages and dendritic cells	SP-D	(60)
Calrecticulin/CD91	Macrophages, neutrophils	SP-A and SP-D	(61, 62)
CD93 (C1qRp)	Endothelial cells, platelets, neutrophils, monocytes, microglial cells, monocytes	SP-A	(63, 64)
CR1	B cells, monocytes, neutrophils, monocytes, microglial cells	SP-A	(65)
SIRPα	Myeloid lineage cells	SP-A and SP-D	(61, 66, 67)
SIRPβ	Myeloid lineage cells	SP-D	(67)
Osteoclast-Associated Receptor (OSCAR)	CCR2+ monocytes	SP-D	(68)
NKp46	NK cells	SP-D	(69)
leukocyte‐associated Ig‐like receptreceptor‐1/2 (LAIR 1/2)	T cells	SP-D	(70)
Fc Receptor γII (FcγRII/CD32)	Eosinophils	SP-D	(71)
TLR2, TLR4 and MD-2	Myeloid lineage cells	SP-A and SP-D	(72–75)
CR3 (CD11b/CD18)	Macrophages	SP-A	(76, 77)
Ig-Hepta (GPR116)	Type II cells	SP-D	(78)
Uroplakin Ia	Bladder epithelial cells	SP-D	(79)
Epidermal Growth Factor Receptor (EGFR)	Human lung adenocarcinoma epithelial cell lines	SP-D	(80)
Gp 340	Macrophages	SP-A and SP-D	(81, 82)
MPO	Neutrophils	SP-A and SP-D	(83)
C1q	Macrophages	SP-A	(84, 85)
Immunoglobulins	Soluble	SP-A and SP-D	(86, 87)
Defensins	Soluble	SP-D	(88, 89)
Decorin	Soluble	SP-D	(90)

#### Clearance of Apoptotic and Necrotic Cells

Promoting the clearance of apoptotic cells before the later stages of apoptosis and necrosis is important in preventing cell membrane breakdown and leakage of toxic intracellular enzymes, which can lead to inflammation and damage to the delicate lung tissue (129, 130). Administration of rfhSP-D is effective in clearing apoptotic and necrotic cells from the lungs of SP-D knockout mice (100). Both SP-A and SP-D bind and enhance the clearance of apoptotic cells, including polymorphonuclear leukocytes (PMNs) and T cells, through distinct mechanisms (131–135). SP-A and SP-D suppress alveolar macrophage phagocytosis through binding of the CRD to signal-regulatory protein (SIRP)α in the resting lung. However, upon initiation of inflammation, SP-A and SP-D activate phagocytosis through binding to the CD91 receptor. Thus, SP-A and SP-D play flexible roles in modulating the inflammatory response depending on the lung environment (61, 66, 136).

#### Removal of Damage-Associated Molecular Patterns (DAMPs) and Neutrophil Extracellular Traps (NETs)

During cell apoptosis, nuclear fragments migrate toward the plasma membrane which form “bleb” like protrusions to display deoxyribonucleic acid (DNA) and ribonucleoproteins at the cell surface (137). One mechanism by which rfhSP-D has been shown to bind to and enhance the clearance of apoptotic cells *in vivo* is through binding genomic DNA (138). SP-D interacts with neutrophil extracellular traps (NET) while simultaneously binding to carbohydrate ligands *in vivo*. Through this mechanism, SP-D agglutinates *Pseudomonas aeruginosa* and alters the mode of NET-mediated bacterial trapping (139). SP-D has also been shown to inhibit eosinophil extracellular DNA trap formation. This effect was lost upon nitrosylation of SP-D, highlighting the potential differing role in modulating eosinophil DNA trap formation depending on the inflammatory status of the lung (140). Alongside binding to apoptotic cells, cell debris and extracellular traps, SP-D binds free bacterial and host DNA. Palaniyar et al. demonstrated the decreased clearance and accumulation of both free DNA and auto-antibodies in SP-A and SP-D knockout mice (141, 142).

#### Clearance of Allergens

Clearance of allergens is also an important function of SP-A and SP-D as allergens may have a synergistic role with viruses in inducing exacerbations of inflammatory lung diseases including asthma (143). SP-A and SP-D are widely reported to bind to and enhance the uptake and clearance of allergens from *A. fumigatus* (144), house dust mite (145) and various types of pollen (146, 147). Furthermore, they modulate the allergen-induced inflammatory response by reducing basophil, eosinophil and mast cell degranulation to prevent the release of pro-inflammatory mediators including histamine and beta-hexosaminidase (106, 158–160, 162). rfhSP-D modulates allergic inflammatory responses and reduces mast cell and basophil degranulation in allergic inflammation *in vivo*. rfhSP-D also both prevents eosinophil recruitment in allergen-challenged mice and enhances the apoptosis and clearance of primed eosinophils by macrophages and PBMCs (148–151).

#### Interaction With Noxious Particles

Inhalation of noxious particles is an important risk factor for the development of inflammatory lung diseases such as chronic obstructive pulmonary disease (COPD), and patients with COPD have an increased risk of viral lower respiratory tract infections (LRTI) (152–154). SP-A and SP-D agglutinate and clear a range of different hydrophobic and hydrophilic nanoparticles and rfhSP-D enhances the co-localization of nanoparticles to epithelial cells *in vitro* (155, 156). Nanoparticles may inhibit the capacity of SP-A and SP-D to neutralize influenza virus (2, 155, 157). Diesel exhaust pollutant exposed mice have an increased susceptibility to influenza and RSV infection, associated with a decrease in surfactant protein expression (157, 158). Thus, both decreased expression and modulation of SP-A’s and SP-D’s anti-viral activity could play roles in the increased susceptibility of smokers and COPD patients to viral LRTI. Moreover, SP-A and SP-D could play additional indirect roles in preventing viral infection and inflammation through the clearance of noxious particles.

### Modulation of the Innate Immune Response by SP-A and SP-D

SP-A and SP-D interact with various receptors on innate immune cells to modulate inflammation (**Table 1**) (119). An elegant model by Gardai et al. demonstrated the dual manner by which SP-A and SP-D mediate or suppress inflammation, dependent on the orientation of the collectin and, therefore, receptor with which it interacts; a similar mechanism to their role in modulating apoptosis (61). Gardai et al. described the interaction of SP-A and SP-D through the CRD with SIRPα on myeloid lineage cells in the resting lung. This was shown to prevent pro-inflammatory cytokine production to maintain homeostasis. However, upon occupation of the CRD through pathogen binding, SP-A and SP-D instead interact with the calrecticulin/CD91 receptor complex through their N-terminal tails. This mediates the production of pro-inflammatory cytokine production for anti-pathogen immune responses. An exemplar of this dual role in viral infection has been demonstrated by the ability of SP-D to decrease neutrophil burst *in vitro*, but increase neutrophil burst in the presence of influenza (159).

SP-A and SP-D bind various other receptors on alveolar macrophages and have been shown to reduce TNF-α production through competing with LPS for CD14 binding (**Table 1**) (160). Furthermore, SP-A and SP-D modulate inflammatory cytokine production after stimulation of macrophages by cytokines or PAMPs. For example, SP-A inhibits peptidoglycan-induced TNF-α secretion upon binding to toll-like receptor (TLR)-2. SP-A also inhibits TNF-α production in IFN-γ stimulated macrophages to reduce nitric oxide production (161, 162). Minutti et al. demonstrated the role of SP-A in directly binding to IFN-γ and inhibiting IFN-γ and LPS–induced TNF-α, inducible nitric oxide synthase (iNOS), and C-X-C motif chemokine ligand 10 (CXCL10) production (163). SP-A and SP-D also mediate alveolar macrophage and neutrophil chemotaxis and stimulate alveolar macrophage directional actin polymerization (164–167).

#### Interactions With Newly Discovered Receptors on Monocytes

Two new receptors have recently been discovered for SP-D on monocytes which demonstrate the dual role SP-D plays in modulating their functions through the collagen domain. SP-D binds to Leukocyte-associated Ig-like receptor-1 (LAIR1), a receptor expressed on neutrophils and monocytes, and prevents the production of FcR-mediated ROS, in a human myeloid leukemia cell (70). However, SP-D also binds to osteoclast-associated receptor (OSCAR) on human C-C chemokine receptor 2 positive (CCR2+) inflammatory monocytes to activate TNF-α release through its collagen domain (68). Further work to investigate the impact of these interactions in anti-viral and inflammatory responses is needed.

#### Interaction With Innate Lymphoid and Natural Killer Cells

Although there has been limited research investigating the interaction of SP-A and SP-D with innate lymphoid cells (ILC), one study demonstrated the importance of SP-D in mediating effective ILC2-mediated immune responses to the parasite *Nippostrongylus brasiliensis* (168). SP-D knockout mice had an impaired ability to resolve *N. brasiliensis* infection. However, intra-nasal treatment with rfhSP-D was shown to increase numbers of IL-13 producing ILC2s and numbers of alternatively activated macrophages in the lung. Moreover, rfhSP-D administration enhanced parasitic killing during the larval L4 lung stage of its natural life cycle (168).

Natural killer (NK) cells are an essential component of the anti-viral immune response. However, little is known about their interaction with SP-A and SP-D. A study by Ge et al. demonstrated a decreased IFN-γ expression in SP-D knockout mice upon ozone exposure (69). The authors hypothesized that this decrease was as a result of the absence of SP-D interacting with the glycosylated NKp46 receptor on NK cells (69). They further postulated that this could play a role in the impaired dendritic cell homing to lymphoid tissue seen in SP-D knockout mice. SP-A has also been suggested to interact with NK cells through the SPR-210 receptor, now identified as Myosin 18A (or CD245) (53). A study looking at the impact of SP-A on NK cell function found an increase in IL-2 activated NK cell-mediated lymphokine-activated killer (LAK) activity toward Epstein-Barr Virus-infected B cells (169). These interactions could have important potential consequences for modulating NK cell function in anti-viral and inflammatory responses. Further work characterizing the role of SP-A and SP-D in NK-cell mediated anti-viral responses may be important in understanding the pathogenicity of emerging viral threats.

### SP-A and SP-D: Orchestrators of the Adaptive Immune System

#### Interaction With Dendritic Cells

SP-A and SP-D bridge the innate and adaptive immune system through their functions in modulating DC function. These roles could be key in directing the inflammatory response after respiratory viral infection. SP-D knockout mice have increased activation of DCs as demonstrated by CD11b and CD86 co-stimulatory molecule upregulation and increased TNF-α expression; this is corrected upon treatment with recombinant murine SP-D (170). Furthermore, SP-A and SP-D modulate lung DC function through inhibiting antigen presentation and SP-A has been shown to inhibit *E. coli* antigen presentation, while simultaneously increasing its phagocytosis (171, 172).

The resultant impact of collectin-modulated DC function on T cells has been demonstrated by a decrease in LPS-mediated major histocompatibility complex II (MHCII) and CD86 expression by DCs and a reduction in allo-stimulation of CD4 T cells upon treatment with SP-A. DCs from SP-D knockout mice also express thymus and activation-regulated chemokine (TARC), which is chemotactic for activated T cells (104).

#### Modulation of T Cell Responses

SP-A and SP-D directly modulate T cells and inhibit antigenic and mitogenic induced T cell proliferation through both IL-2-dependent and IL-2-independent mechanisms (173–178). They also alter T cell function and activation. For example, SP-D knockout mice have increased numbers of activated CD4 and CD8 T cells which express CD69 and CD25, while SP-A has been shown to reduce IFN-γ production and T cell mediated inflammation (101, 104, 178). Treatment with rfhSP-D both decreases T cell activation and lymphoproliferation through the upregulation of cytotoxic T-lymphocyte-associated protein 4 (CTLA4), as well as decreasing allergen induced IgE production by B cells (148, 179).

SP-D impacts the adaptive immune system by modulating T cell apoptosis. This is seen through the prevention of caspase-8 and caspase-3 activation by SP-D to inhibit Fas (CD95)-Fas ligand and tumor necrosis factor-related apoptosis-inducing ligand (TRAIL)-TRAIL receptor induced apoptosis (180, 181). T cells are essential in anti-viral immunity and collectins could play roles in coordinating this response (182). Further investigation of the roles of SP-A and SP-D in modulating T cell-mediated responses and viral-mediated immunopathology is now indicated, through the use of appropriate *ex vivo* viral and inflammatory models (183).

## Deficiency of Collectins in Inflammatory Lung Diseases

Consistent with their array of roles in viral immunity and lung homeostasis, SP-A and SP-D deficiency may contribute to pathological mechanisms in a range of respiratory diseases. Winkler et al. demonstrated a decreased level of SP-D in the airways of smokers, with a further reduction in patients with COPD (2). This was inversely related to serum SP-D levels due to leakage of alveolar SP-D across the inflamed or damaged alveolar capillary membrane. Patients experiencing COPD exacerbations have higher serum SP-D levels which are reduced by treatment with anti-inflammatory glucocorticoids, highlighting its potential role as a biomarker for COPD acute inflammation (184, 185). SP-A and SP-D have also been shown to be reduced in allergen-challenged asthma patients and lower airway levels have been shown to correlate with asthma severity (1, 186).

Along with lipid surfactant, SP-A and SP-D levels are deficient in premature neonates. However, these key proteins are not replaced with current surfactant therapy, as they are not present in current formulations. Low collectin levels are correlated with risk of infection and development of neonatal chronic lung disease, both in animal models of preterm lung disease and in clinical studies. In particular, low SP-D levels in human preterm infants soon after birth are linked with an increased risk of neonatal chronic lung disease development (3, 187–189). SP-D levels increase in response to infection in the preterm infant, but this acute phase response may be inadequate to counter ongoing inflammation, due to degradation in the inflamed preterm lung. Exogenous therapeutic recombinant SP-D administration has been shown to reduce ventilation-induced inflammation in preterm lambs, highlighting its potential to reduce inflammation caused by barotrauma in ventilated preterm infants developing neonatal chronic lung disease (190, 191).

**Figure 3:**
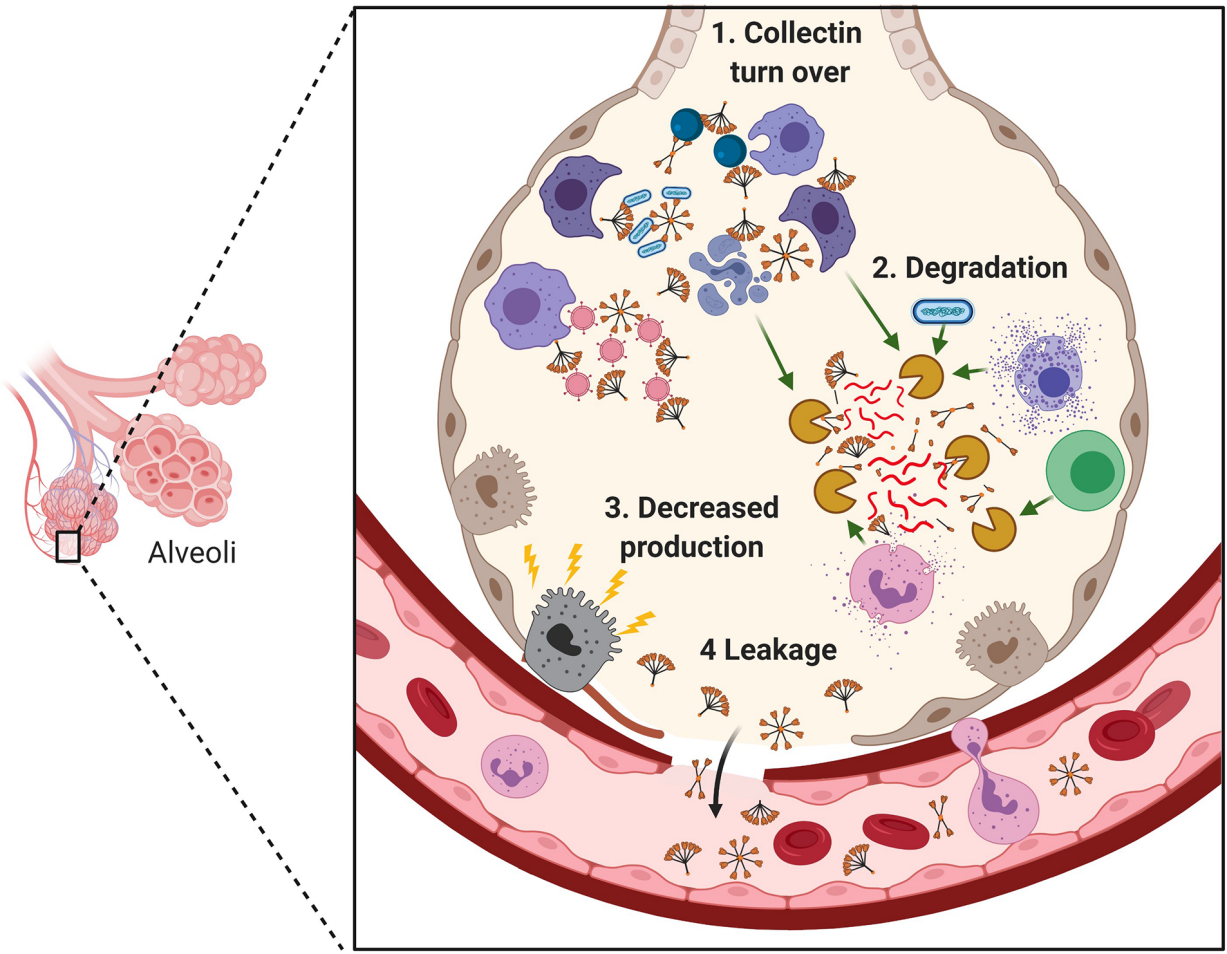
Mechanisms for reduction in surfactant proteins A (SP-A) and SP-D in the lung during infection and inflammation. Indicated is the degradation of SP-A and SP-D through their role as scavenger receptors to bind and enhance clearance of pathogens, noxious particles, apoptotic cells and cell debris (1); degradation of SP-A and SP-D through pathogen-derived proteases and elevated endogenous proteases secreted by recruited inflammatory cells or released from dying and damaged cells (2); damage to the alveolar epithelium leading to reduction of SP-A and SP-D production (3) and leakage into the blood (4). Adapted from “Cytokine Storm”, by BioRender.com (2020). Retrieved from https://app.biorender.com/biorender-templates.

Deficiency of collectins in inflammatory lung diseases could be related to multiple factors (**Figure 3**). Firstly, SP-A and D have key roles as scavenger molecules in maintaining lung homeostasis. Thus, in the chronic inflammatory environment of the diseased lung, a constant turnover and degradation of SP-A and SP-D through binding to and enhancing clearance of pathogens, noxious particles, apoptotic cells and cell debris could lead to decreased levels. Furthermore, inflammatory mediators which damage the delicate epithelium could compromise the air-blood barrier with resultant leakage of SP-A and SP-D from the lung into the blood. Alveolar type 2 cell injury could similarly lead to a reduction in SP-A and SP-D lung levels due to decreased synthesis (192).

Degradation of SP-A and SP-D through pathogen-derived proteases and elevated endogenous proteases, secreted by recruited inflammatory cells or released from dying and damaged cells, may also play a role in reducing SP-A and SP-D levels within the inflamed lung. SP-A and SP-D are degraded through various host and pathogen-derived enzymes including leucocyte elastase, proteinase 3, cathepsin G and Pseudomonas elastase (193–196). Children with cystic fibrosis have protease - antiprotease imbalance as well as coexisting low levels of SP-A and SP-D (197–199). Decreased SP-A and SP-D levels have also been found in BAL from children with RSV infection (21). Low SP-A and SP-D levels in such inflammatory lung diseases may both generate susceptibility to respiratory viral infection and lead to an exaggerated damaging host inflammatory response.

## Discussion

The potential for treatment of inflammatory diseases and respiratory viral infections by augmentation of the innate immune system is increasingly understood but as yet remains unexploited (200–202). SP-A and SP-D are anti-viral innate immune molecules and play key roles in orchestrating the innate and adaptive immune system to limit inflammation, making these mechanisms dually attractive as potential therapeutics.

Correction of SP-A and SP-D deficiency in inflammatory respiratory diseases could be achieved by supplementation with recombinant versions of SP-A and SP-D. However, development of full-length recombinant SP-A and SP-D molecules as therapeutics has been problematic due to low expression yields in eukaryotic systems. Furthermore, difficulties with handling of the proteins, with a tendency to oligomerize and/or agglomerate, generates difficulties with precise stable molecular characterization and solubility (203–208).

Smaller rfhSP-A and rfhSP-D trimeric fragment proteins have been developed and have the advantage of being more easily and cheaply produced in *E. coli* (209–211). These proteins maintain many of the anti-viral and immunomodulatory functions of the native proteins (11–13, 15, 45, 46, 212). Furthermore, they contain the functional CRD binding domain which mediates the anti-inflammatory collectin action through interacting with SIRPα on innate immune cells (61). However, they lack the majority of the collagen domain and the N-terminus of the native full-length protein. The N-terminal region has been shown to induce inflammation through binding calrecticulin/CD91 and may be exploited to facilitate a route of entry for viruses such as HIV, RSV, and the Ebola virus (14, 96, 61).

rfhSP-D functions to neutralize RSV, HIV and SARS-CoV-2 and modulates influenza-mediated inflammatory cytokine production (25, 45, 46, 53, 52). rfhSP-D also corrects the emphysematous phenotype seen in murine SP-D deficiency and reduces levels of apoptotic and necrotic macrophages and MMPs. rfhSP-D binds to and enhances the clearance of apoptotic cells, free DNA and neutrophil and eosinophil extracellular traps (139, 140) and modulates the adaptive immune system to suppress proliferation and activation of T cells through upregulation of CTLA4 (101, 179, 213). rfhSP-D, therefore, has properties which suggest it may be useful both as a prophylactic and treatment for infectious and inflammatory lung diseases. rfhSP-A has been shown to neutralize RSV, but requires further characterization (11).

Therapeutic rfhSP-D is currently under development for treatment of premature neonates with neonatal RDS as an adjunct to current surfactant therapies to help prevent the development of chronic inflammation leading to neonatal chronic lung disease. This may help prevent the inflammatory emphysematous phenotype seen in neonatal chronic lung disease and reduce susceptibility to severe respiratory viral infection. Alongside other inflammatory diseases such as asthma and COPD, rfhSP-D could have therapeutic potential in emerging respiratory infections such as SARS-CoV-2, by both neutralizing the virus and modulating the inflammation-mediated pathology associated with COVID-19.

## Author Contributions

AW: conceptualization, investigation, literature searching, analysis, project administration, writing original draft, reviewing and editing. JM: supervision, conceptualization, reviewing, and editing. HC: conceptualization, funding, supervision, writing, reviewing, and editing. All authors contributed to the article and approved the submitted version.

## Funding

This work was supported by the UK Medical Research Council (*MR/P026907/1)* and by the UCLH/UCL NIHR Biomedical Research Centre.

## Conflict of Interest

AW, JM, and HC are named inventors on a patent jointly filed by University of Southampton and Spiber Technologies (WO2017109477A2·2017-06-29).
